# Recanalization of the PRESERFLO MicroShunt With a 9-0 Nylon Suture: A Case Report

**DOI:** 10.7759/cureus.99452

**Published:** 2025-12-17

**Authors:** Mao Ishiyama, Yuta Kitamura, Tomoaki Tatsumi, Takayuki Baba

**Affiliations:** 1 Department of Ophthalmology and Visual Science, Chiba University Graduate School of Medicine, Chiba, JPN; 2 Department of Ophthalmology, Inoue Memorial Hospital, Chiba, JPN

**Keywords:** as-oct, elevated intraocular pressure, glaucoma, intraocular pressure, preserflo microshunt, surgical complication

## Abstract

Clinical studies have evaluated the safety and efficacy of PRESERFLO MicroShunt (PMS) (Santen Pharmaceutical, Osaka, Japan) implantation, suggesting that this surgery is associated with fewer serious complications and significant intraocular pressure (IOP) reduction. Although there have been reports of iris obstruction at the tip of the tube, no cases of luminal occlusion at the mid-portion of the tube or its management have been reported.

This case report describes a 57-year-old man who underwent PMS for primary open-angle glaucoma (POAG) in the right eye. One month after surgery, the IOP in the right eye had increased to nearly 40 mmHg. Initial needling around the distal end of the tube was attempted but proved ineffective. We then examined the tube lumen using anterior segment optical coherence tomography (AS-OCT), which revealed a suspected luminal obstruction at the center of the tube. Upon removal of the tube, intraluminal tissue debris was found at the center of the lumen. A 9-0 nylon suture was inserted into the lumen to dislodge and remove the debris, after which the tube was reinserted. Postoperatively, a well-formed filtration bleb was observed, and the IOP decreased to 8 mmHg. One year after surgery, the IOP in the right eye remained stable at 12 mmHg, with no evidence of re-occlusion of the tube.

The insertion of a 9-0 nylon suture may be an effective method for clearing luminal debris in PMS tubes. In addition, AS-OCT is a useful tool for detecting tube lumen obstructions.

## Introduction

Glaucoma is a degenerative optic nerve disease caused by retinal ganglion cell death associated with optic nerve axon degeneration, resulting in chronic progressive visual field defects. It is a leading cause of irreversible blindness worldwide, and its prevalence is increasing [1]. Currently, lowering the intraocular pressure (IOP) is the only evidence-based treatment available to control glaucoma progression. Although medication and laser therapy are available as treatment options to lower IOP, there are cases in which IOP reduction is insufficient, and surgical IOP reduction is sometimes required. Recently, a new filtration surgery technique using the PRESERFLO MicroShunt (PMS) (Santen Pharmaceutical, Osaka, Japan) has attracted considerable attention. This device functions by creating a permanent conduit for aqueous humor to flow from the anterior chamber to the sub-Tenon's space (the potential space between Tenon's capsule and the sclera), bypassing the resistant trabecular meshwork. This shunt device is 8.5 mm long, 350 µm in outer diameter, and 70 µm in lumen diameter, dimensions specifically designed to regulate aqueous flow according to the Hagen-Poiseuille equation, lower IOP, and prevent occlusion [2,3]. In vitro experimental results showed PMS has the potential to significantly lower intraocular pressure (IOP) to approximately 2.6 mmHg [4]. The PMS is made of poly(styrene-block-isobutylene-block-styrene), also known as SIBS, a highly biocompatible and bioinert material that can resist foreign body reactions, inflammation, scarring, and encapsulation of filtering blebs [3]. The posterior end is positioned approximately 3 mm from the insertion site on the fornix side to avoid contact with the eyelids. In recent years, several clinical studies have evaluated the safety and efficacy of PMS implantation, suggesting that this surgery is associated with fewer serious complications and significant IOP reduction [5-8]. In addition, data have demonstrated non-inferiority in IOP-lowering efficacy and safety compared with trabeculectomy, a conventional filtration surgery [9-11]. Adverse events reported with this procedure include hyphema, transient postoperative hypotony, transient choroidal effusion or detachment, shallow or flat anterior chamber, iris touching devices, corneal touching devices, and tube exposure [5,12-14]. Although there have been reports of iris obstruction at the tip of the tube, there have been no reports of lumen occlusion near the center of the tube or the management thereof. We aimed to report a case wherein black tissue debris was trapped in the lumen of the tube, resulting in severe IOP elevation in the early postoperative period; we used a 9-0 nylon suture to successfully remove the intraluminal tissue debris and restore filtration after reuse.

## Case presentation

A 57-year-old male patient with open-angle glaucoma was referred to our hospital with uncontrollable IOP in both eyes with maximal glaucoma therapy. He had no other eye diseases and no history of ocular surgery, but had undergone selective laser trabeculoplasty (SLT) twice in the left eye because of high IOP at the clinic one week prior to presentation. He had no history of systemic diseases, steroid treatment, or ocular trauma. He had smoked 20 cigarettes per day for 37 years. The patient had no family history of glaucoma. The patient used three glaucoma eye drops (latanoprost 50 mg/mL once a day, brinzolamide/timolol 10 mg/mL+5 mg/mL twice a day, and ripasudil 4 mg/mL twice a day), and oral acetazolamide 500 mg twice a day was administered.

At the first presentation, the best-corrected visual acuity was 1.2 in the right eye and 1.0 in the left eye. The spherical power was -1.5 D in the right eye and -1.0 D in the left eye. The baseline preoperative IOP on applanation Goldmann tonometry at the first visit was 36 mmHg in the right eye and 26 mmHg in the left. Slit-lamp examination revealed bilateral deep anterior chambers with no evidence of inflammation, pseudoexfoliation, phacodonesis, or pigment dispersion. Gonioscopy revealed an open-angle condition with no evidence of peripheral anterior synechia or neovascularization. Funduscopic examination revealed enlarged disc cupping, predominantly in the right eye, and no other pathological findings in the macula or peripheral retina. OCT revealed thinning of the inferior ganglion cell complex (GCC) layer in the right eye and a positive temporal raphe sign. No obvious thinning of the GCC was observed in the left eye. Humphrey 30-2 visual field testing revealed a superior visual field defect in the right eye, but no apparent glaucomatous visual field defects in the left. The patient was diagnosed with primary open-angle glaucoma (POAG) of the right eye and ocular hypertension of the left eye.

**Figure 1:**
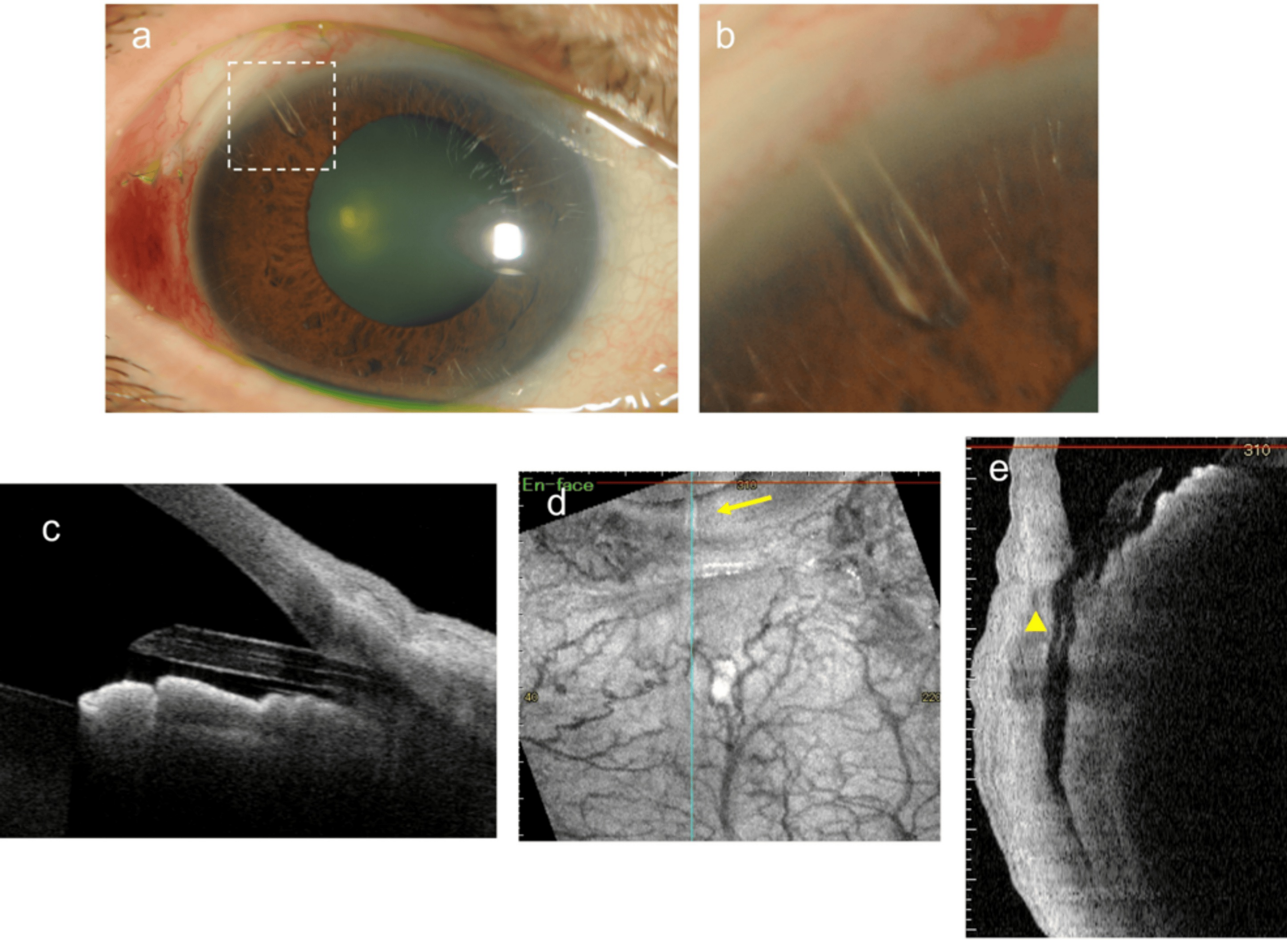
Findings one month after PRESERFLO MicroShunt (PMS) surgery in the right eye (a) Slit-lamp examination showing that the tube edge is not in contact with the iris. (b) Magnified view of the area enclosed by the dotted line in 1a. (c) Anterior segment optical coherence tomography (AS-OCT) image of the anterior chamber angle showing that the edge of the tube is not in contact with the iris. (d) En-face optical coherence tomography (OCT) imaging of blebs. The yellow arrow indicates the edge of the PMS tube in the anterior chamber. (e) Corresponding vertical cross-sectional image. The yellow arrowhead indicates the high-intensity reflection in the lumen near the center of the tube.

**Figure 2:**
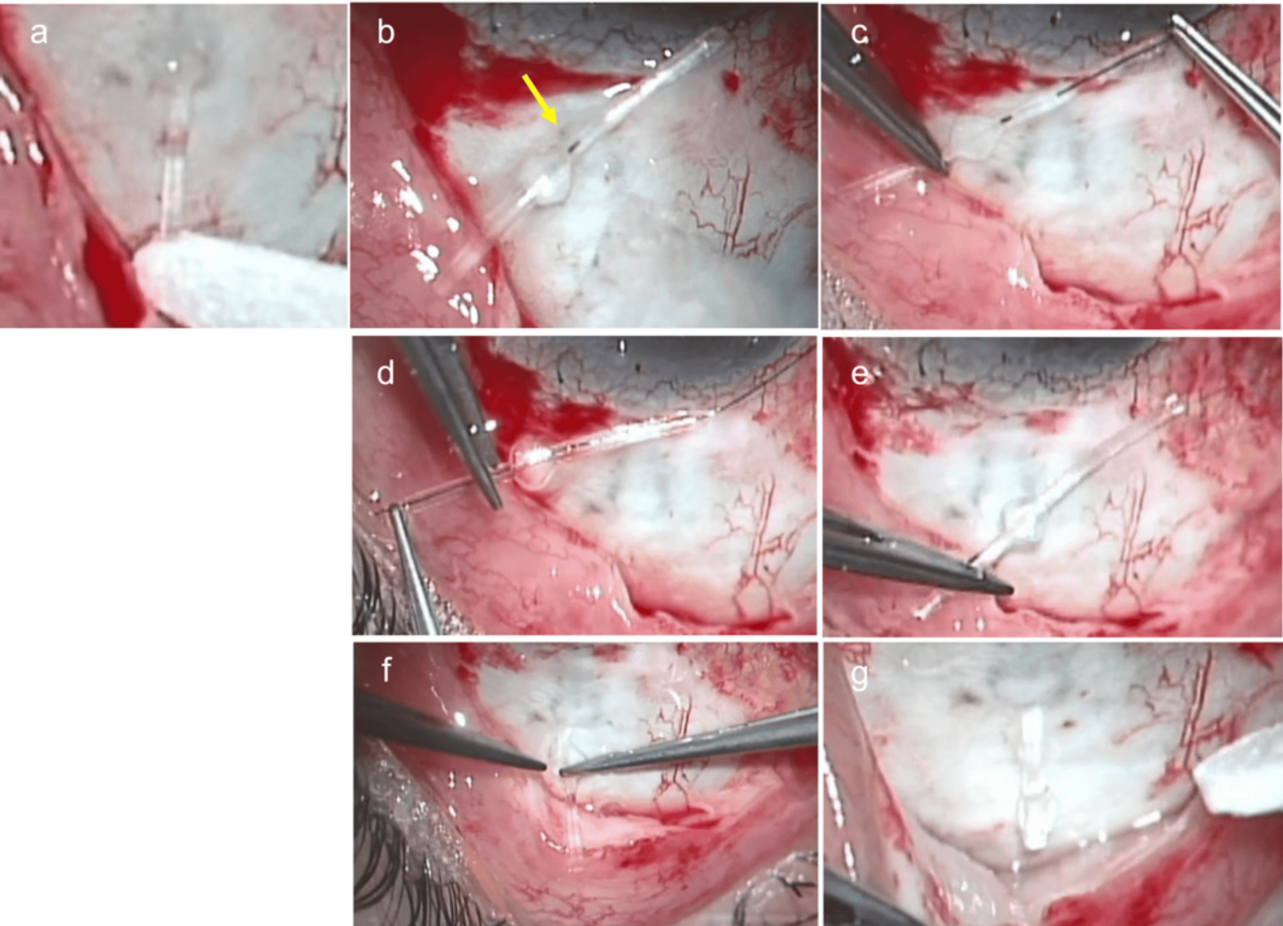
Images at the time of reoperation (a) The exposed PRESERFLO MicroShunt (PMS) tube inserted during the initial surgery. There was little aqueous outflow from the end of the tube even after performing ocular massage and flushing with balanced saline solution (BSS). (b) The tube after removal. Black tissue debris (arrow) were trapped in the lumen near the anterior central fin of the tube. (c-d) Image of 9-0 nylon suture being inserted with microforceps to remove debris in the tube lumen. (e) Image after removing debris in the lumen of the tube by passing a 9-0 nylon suture and cleaning with BSS. (f) Image of the tube being reinserted into the previously created scleral tunnel. (g) Image after completion of tube reinsertion. Aqueous outflow from the tip of the tube was observed.

**Figure 3:**
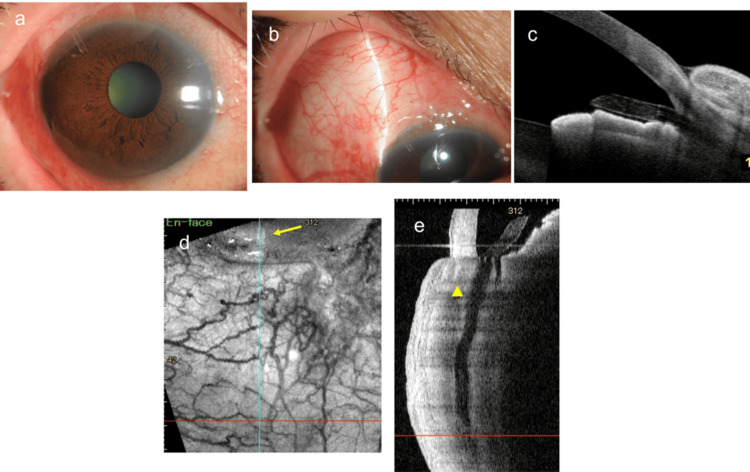
Findings at one month after reoperation (a) Slit-lamp image. (b) Slit-lamp image of the bleb section. A well-filtered bleb was observed. (c) Anterior segment optical coherence tomography (AS-OCT) image of the anterior chamber angle. (d) En-face OCT image of the bleb. The yellow arrow indicates the edge of the PRESERFLO MicroShunt (PMS) tube in the anterior chamber. (e) Corresponding vertical cross-sectional image. The preoperative hyperintense reflection in the lumen near the center of the tube has disappeared (yellow arrowhead).

Owing to poor IOP control with medical therapy, PMS surgery with 0.04% MMC was performed in the right eye. After creating a scleral tunnel and performing an anterior chamber puncture, the PMS tube was successfully inserted at the third attempt. Although aqueous humor outflow from the tip of the tube was slower than usual, it was restored after the intraluminal tube was washed with balanced salt solution (BSS) several times. Further procedures were completed without further complications. The IOP decreased significantly to 16 mmHg on the day after surgery and was lowered to 6 mmHg with ocular massage. No bleeding or fibrin formation was observed in the anterior chamber postoperatively. Thereafter, although it remained in the mid-10 mmHg range without glaucomatous eye drops, at the follow-up visit in the first postoperative month, the IOP in the right eye increased to 39 mmHg at one month after surgery. Since the edge of the tube in the anterior chamber was open and no contact with the iris was observed (Figures 1a-1c), we initially suspected that the high IOP was caused by the progressive growth of connective tissue or obstruction by Tenon’s tissue at the distal end of the tube. Therefore, we attempted needling with a 27-G needle to release the obstruction around the tube’s end. However, the postoperative IOP was still high at approximately 30 mmHg. To visualize the lumen of the MicroShunt, we used AS-OCT. Observation of the running area of the tube revealed high-intensity reflections in the lumen near the center of the tube (Figures 1d-1e). Considering the possibility of increased IOP due to occlusion of the tube lumen, we decided to perform a bleb revision surgery. We flushed the lumen of the tube with balanced saline solution (BSS) several times, but there was little aqueous outflow from the MicroShunt (Figure 2a). When the tube was removed, black tissue debris was trapped in the lumen near the anterior portion of the central fin of the tube (Figure 2b). To remove the intraluminal tissue debris, we first tried threading the lumen of the tube with 10-0 nylon suture, but were unable to remove the debris. Next, a 9-0 nylon suture was inserted through the tube with microforceps (Figures 2c-2d), and the lumen was further cleaned with BSS, which confirmed that the debris had been removed completely (Figure 2e). The tube with the cleaned lumen was reinserted into the previously created scleral tunnel (Figure 2f), and good aqueous outflow was observed from the MicroShunt (Figure 2g). One month later, the IOP was 8 mmHg, bleb formation was well maintained, and the tube was well-positioned (Figures 3a-3c). AS-OCT observation of the tubes showed that the luminal hyperintense reflections observed preoperatively had disappeared (Figures 3d-3e). One year after reoperation, the right IOP was 12 mmHg, and the bleb formation was well-maintained.

## Discussion

This case demonstrates a rare postoperative complication in which the lumen of the PMS tube was occluded near the center. Intraluminal tissue debris was removed by inserting a 9-0 nylon suture into the tube and restoring filtration upon reuse. Since there have been no reported cases of intraluminal debris entering the center of the lumen and causing obstruction after PMS surgery, either in clinical trial results or case reports, the frequency of such cases is unknown. There is a report of a 10-0 nylon suture being used to release a tube obstruction after surgery with a XEN45 gel stent [15]. In the present case, a 10-0 nylon thread was attempted, but it was not sufficient to remove the obstruction. Since the inner diameter of the tube is slightly wider in PMS (70 μm) than in the XEN45 gel stent (45 μm), a 9-0 nylon with a diameter of approximately 35 μm may be more appropriate than a 10-0 nylon with a diameter of approximately 25 μm to remove intraluminal debris in the case of PMS. The mechanism of how and when the foreign body entered the lumen in this case is difficult to explain precisely. When evaluating elevated IOP in the early postoperative period following PMS implantation, differential diagnoses typically include obstruction of the tube tip by the iris, blockage by blood or fibrin clots, or early encapsulation. There have been previous reports of occlusion due to contact between the tube tip and iris [12,14]; however, in this case, no evidence of contact between the iris and tube tip was observed on slit-lamp examination or AS-OCT postoperatively. Moreover, at the end of the surgery, the anterior chamber was deeply maintained, and the tube was not in contact with the iris. Therefore, occlusion due to contact with the iris is unlikely. Because there was an episode during PMS surgery in which insertion of the tube was difficult the first and second times because of resistance in the middle of the insertion, the tube was removed and inserted repeatedly, and the tube finally reached the anterior chamber on the third insertion. While a manufacturing defect of the lumen could theoretically cause obstruction, the presence of aqueous outflow immediately upon final insertion suggests the lumen was patent at the time of manufacture. Considering these facts, it is highly probable that some tissue remaining in the scleral tunnel (suspected uveal or trabecular meshwork tissue) was 'cored' or trapped in the lumen during the repeated insertion attempts against resistance. Since aqueous humor outflow was observed from the terminal portion of the tube immediately after insertion, we did not perform an intraoperative check for the presence of foreign material in the lumen of the tube. However, because the IOP on the postoperative day was relatively high (16 mmHg), it was expected that the foreign material was already trapped, albeit incompletely, in the tube lumen during surgery. One explanation for this is that the intraluminal debris, which had been dispersed to some extent throughout the lumen of the tube, may have accumulated in one place over time and formed a large mass, obstructing the lumen of the tube and increasing the outflow resistance. In the present case, AS-OCT was performed to observe the tube inside the scleral tunnel. We detected high reflectivity in a part of the lumen, suggesting occlusion due to the presence of a foreign body. Some reports have indicated the usefulness of AS-OCT to observe the inside of a glaucoma drainage device (GDD) tube, suggesting that AS-OCT may also be useful in PMS to evaluate tube lumen occlusion or tube exposure.

Blood clots, fibrin, vitreous humor, and contact with the iris have been identified as causes of tube occlusion. In addition to surgical removal of the obstruction with forceps or scissors, there are reports of the effectiveness of the Nd: YAG laser at the edge of the tube when the obstruction is caused by a blood clot, fibrin, or iris [16-18]. In PMS, complications of iris occlusion have been reported in approximately 2-13% of cases [8], and in most cases, no additional treatment is required; however, there have been reports of removal of obstructing iris fibers with retinal scissors and forceps [12] and one case of laser therapy [14]. If, as in this case, the occlusion is located at the center of the lumen of the tube rather than at the opening, the tube must be removed. In the present case, we removed the intraluminal debris by passing a 9-0 nylon suture through the lumen of the tube and reinserting the tube into a previously created scleral tunnel, which showed good aqueous outflow and reduced postoperative IOP. Although it remains to be determined whether this method is effective in all occlusion cases, it appears to be an effective option for occlusion removal. Inserting a 9-0 nylon suture from the anterior chamber into the lumen of the tube without incising the conjunctival tissue could be considered; however, this may be an issue for future consideration because the inner diameter of the MicroShunt tube is quite small, making it difficult to manipulate the suture. Finally, if the MicroShunt tube is difficult to insert and requires repeated insertion, and if aqueous flow from the tip of the tube is slower than normal, it may be better to suspect an intraluminal obstruction and check it once.

## Conclusions

In conclusion, this case demonstrated a rare complication of PMS-IOP elevation due to intraluminal occlusion of tissue debris early after surgery. This case also demonstrates the efficacy of AS-OCT in detecting occlusion of the MicroShunt tube and the utility of the 9-0 nylon suture in removing intraluminal debris. However, further studies are needed to confirm this hypothesis.
